# Poor Law Topics

**Published:** 1906-01-27

**Authors:** 


### POOR LAW TOPICS.

### THE INDUSTRIAL TRAINING OF CHILDREN.

In many poor-law schools the children are trained for different occupations, and a modification of the "half-time" system is in vogue which can be better carried out in an institution where all the pupils are under the same roof and the same control than in the outer world. In a poor-law school also the wage-earning side of the work is kept in proper abeyance to the educational, and the Guardians as a rule are more keen upon the children being properly taught than on saving a few pounds by getting them to do work which otherwise has to be paid for. So far as the training of the children is concerned all is satisfactory. Girls are with very few exceptions sent into domestic service, and it says much for the training given that "union" girls are in such demand that the matrons of the schools can exercise a scrupulous care as to the situations where they place them. It may be, indeed, that almost too much care is exercised to put the girls in good places—places where they will have so little anxiety, such abundant food, such comfortable conditions that—added to the fact that the girls have been brought up in an institution where food, clothing and all the necessaries of life seem to "come by nature"—their capacity for dealing with the emergencies of life, such as they will meet with when they marry, is never developed. Still the fact remains that there is never any difficulty in placing them satisfactorily. But with boys it is different. When it is time for them to leave the school it is desired to apprentice them to the trade which they have already been taught. The Guardians pay a fee to the master, who must in return take the boy into his house and continue to teach him the trade until he is a competent journeyman. But in many businesses the masters no longer take resident apprentices, and difficulties are found in placing boys in really good shops. The impecunious employer, to whom the instalments of the premium come as a godsend, is often unable to give the boy proper food and lodging, and the work done is not really a continuation of what the boy has already learned; often, indeed, it is only patching, cobbling, and mending of a comparatively unskilled kind. The modern tendency to concentrate industries into large factories is distinctly against the apprentice system, and poor-law authorities have not yet been able to bring their methods into line with the developments of the time. The conditions of labour vary so much in different localities that it is hardly possible for any pronouncement from the central authority to meet all the difficulties of the question, but it should surely be possible for a reputable employer to take a parish boy as apprentice on condition that he placed him in a home which the Guardians would approve, although that home might not be under his own roof. Children so placed would be in much the same position as other boarded-out children, and to release the employer from the necessity of having the apprentice in his own house would facilitate their entrance into many branches of industry from which they are now excluded.

